# DNA Barcoding of Marine Copepods: Assessment of Analytical Approaches to Species Identification

**DOI:** 10.1371/currents.tol.cdf8b74881f87e3b01d56b43791626d2

**Published:** 2014-06-23

**Authors:** Leocadio Blanco-Bercial, Astrid Cornils, Nancy Copley, Ann Bucklin

**Affiliations:** University of Connecticut, Groton, Connecticut, USA; Alfred-Wegener-Institut Helmholtz-Zentrum für Polar- und Meeresforschung, Bremerhavn, Germany; Woods Hole Oceanographic Institution, Woods Hole, Massachusetts, USA; University of Connecticut, Groton, Connecticut, USA

## Abstract

More than 2,500 species of copepods (Class Maxillopoda; Subclass Copepoda) occur in the marine planktonic environment. The exceptional morphological conservation of the group, with numerous sibling species groups, makes the identification of species challenging, even for expert taxonomists. Molecular approaches to species identification have allowed rapid detection, discrimination, and identification of species based on DNA sequencing of single specimens and environmental samples. Despite the recent development of diverse genetic and genomic markers, the barcode region of the mitochondrial cytochrome c oxidase subunit I (COI) gene remains a useful and – in some cases – unequaled diagnostic character for species-level identification of copepods. This study reports 800 new barcode sequences for 63 copepod species not included in any previous study and examines the reliability and resolution of diverse statistical approaches to species identification based upon a dataset of 1,381 barcode sequences for 195 copepod species. We explore the impact of missing data (i.e., species not represented in the barcode database) on the accuracy and reliability of species identifications. Among the tested approaches, the best close match analysis resulted in accurate identification of all individuals to species, with no errors (false positives), and out-performed automated tree-based or BLAST based analyses. This comparative analysis yields new understanding of the strengths and weaknesses of DNA barcoding and confirms the value of DNA barcodes for species identification of copepods, including both individual specimens and bulk samples. Continued integrative morphological-molecular taxonomic analysis is needed to produce a taxonomically-comprehensive database of barcode sequences for all species of marine copepods.

## Introduction

Marine copepods represent a predominant component of the zooplankton throughout the world oceans in both abundance and biomass 1 ^,^ 2. There are more than 2,500 described species of planktonic marine copepods, with species distributions ranging from shallow, brackish, estuarine waters to deep ocean (abysso- and hadopelagic) zones 3. Copepods exhibit a wide variety of biogeographical patterns, from very limited distributions to cosmopolitan and global-ocean ones.

Their high species diversity, together with their relatively small size and apparent similarity among different forms, has made the morphological identification and quantification of copepod species a challenging task 4. In addition, it is likely that there are large numbers of cryptic species within what are now considered recognized species, especially for geographically-widespread taxa 5 ^,^ 6 ^,^ 7.

Considerable effort has been focused on the development and use of genetic approaches to identifying and discriminating marine species in the past ~20 years (reviewed by Bucklin et al. 8). Use of a fragment of the cytochrome* c* oxidase subunit I (COI) gene for discrimination and identification of animal species, i.e., DNA barcoding 9 ^,^ 10, has moved rapidly from novelty to widespread use, although it has not been free of controversy. Objections have focused on uses of barcodes beyond the original intent as a species assignment tool, including DNA taxonomy 11 ^,^ 12, ecological assessment 13, and species discovery 14. Recent improvements in methods for statistical analysis of barcode data 13 ^,^ 15 ^,^ 16 ^,^ 17 and growing focus on the appropriate use and limitations of barcode analysis 18 are advancing the field of DNA barcoding.

Recent DNA barcoding studies of marine planktonic copepods have focused on examination of species-level diversity in particular regions of the ocean 19 ^,^ 20 ^,^ 21 ^,^ 22, and also on particular - usually problematical - taxa 23 ^,^ 24 ^,^ 25 ^,^ 26 ^,^ 27 ^,^ 28 ^,^ 29. Other studies have used DNA barcodes for biogeographical or phylogeographical analyses 30 ^,^ 31 ^,^ 32 ^,^ 33 ^,^ 34. A number of studies have revealed cryptic species 5 ^,^ 33 ^,^ 35 ^,^ 36 ^,^ 37.

This study provides 800 new barcode sequences for 63 copepod species not included in previous studies. These new barcoding records increase both the depth of sampling and also the geographical coverage of existing records, and continue progress toward a taxonomically-comprehensive database or library of DNA barcode sequences for all species of the groups or lineages of interest. Importantly, this study examines a variety of statistical and analytical approaches used for barcode data, and provides new information about the strengths, weaknesses and limitations of DNA barcodes for discrimination and identification of copepod species. A particular focus of this study is the impact of any missing data (i.e., species not represented in the barcode database) on the accuracy and reliability of species identifications. Finally, we offer new guidance and a conceptual framework for continued barcoding efforts to meet challenges of species identification of copepods, one of the most ecologically important and systematically complex groups of marine zooplankton.

## Methods

**Samples analyzed**

Sequences of the COI barcoding region 38 were determined for identified individual specimens collected from various sources from 1992 to 2011, and archived at the University of New Hampshire (1992-2005) or the University of Connecticut (2005-2011). All specimen and collection metadata are included in the GenBank entries. Appropriate reference is made to previously published sequence data. Laboratory protocols (DNA purification, PCR amplification, and sequencing) are as described in previous publications by the authors 20 ^,^ 35.

**DNA sequence data analysis**

Sequences were analyzed using the Molecular Evolutionary Genetic Analysis (MEGA) Ver. 5 ** 39. Sequences were aligned using ClustalW 40, as implemented in MEGA, using the corresponding amino acid translated version. This procedure allows better resolution by removing gap ambiguities, ensures designation of the correct codon reading frame, and minimizes risks of including nuclear pseudogenes with mitochondrial origins, known as *numts* 41 ^,^ 42. Initial tree runs were used to check for very divergent sequences (i.e., potential *numts*), which were removed prior to analysis. A total of eight individual sequences clustered in a single, highly supported, independent clade, that comprised a mixture of species from different orders (two) and families (six) of the Subclass Copepoda (five Calanoida and three Harpacticoida). When compared with the final number (1381 COI sequences) this value can be considered low, although it is necessary to recognize that these eight sequences were those that had passed all initial filters (for example, they did not code for a stop codon, or were extremely aberrant when translated into their correspondent amino-acid sequence).

**Descriptive statistics**

Three different alignments were prepared for analysis in order to study the influence and possible bias due to variation in sequence length and heterogeneity in levels of sequence divergence along the barcode region. The analyzed alignments will be referred to as follows: 1) original alignment, including all 1,381 sequences of any length; 2) standard barcode alignment, including only sequences of >500 bp (see Barcode of Life, http://www.barcodeoflife.org/); and 3) unique barcode alignment, considering only a 400 bp portion (positions 96 to 497) of the barcode region and including only a single copy of all the different haplotypes for each species (576 sequences in all). The unique alignment was subjected to a sliding window analysis of nucleotide diversity (π) using DnaSP Ver. 5 43. Two runs were performed with 10 bp step size and window lengths of 10 bp and 100 bp; results were compared to visualize differences in π along the analyzed region.

Genetic distances within species, genera, families, and orders and between orders were calculated in MEGA using the Kimura 2-parameter (K2P) model 44 for each of the three alignments previously described. Mann-Whitney U tests were carried out based upon the unique alignment distances matrix to compare distances within versus between species and between taxonomic levels. Although K2P was the second-best fit for the dataset (the best corresponded to GTR+I+ Γ), this model was used to allow direct comparison with previously published barcoding studies, which most frequently used this metric 8, despite growing criticism of this metric for barcode analysis 45 ^,^ 46. On the other hand, the same studies have shown that the choice of evolutionary model does not affect success rates of species identifications 45 ^,^ 46; uncorrected *p*-distances perform equally to any model; but see Fregin et al. 47.

**Barcoding resolution**

Initially, two automated statistical techniques for barcoding approaches to species identity assignment were evaluated: 1) automated identification of significant clades after tree reconstruction; and 2) genetic distance-based assignment by the Basic Local Alignment Search Tool (BLAST) method 48. Parallel analyses were carried out on the three alignments. In addition, a non-automated technique for species assignment was considered: the “best close match” 49 combines best match criteria and maximum within-species distance thresholds. Similar to the BLAST approach, this technique analyzes each query individually and identifies the closest sequence within a flexible threshold adapted to each dataset. Although computationally intensive and potentially time-consuming, this approach has been shown to out-perform automated and much more complicated methods 16, especially when the sequences are highly variable and many species are represented by one or a few sequences.

Neighbor-Joining (NJ) trees 50 were reconstructed in MEGA using the K2P evolutionary model for the standard and unique alignments. Maximum Likelihood (ML) phylogenetic tree analyses were done using RAxML Ver. 7.2.8 51 under the GTR+I+Γ model for the three datasets. This model-based method (ML) allows inclusion of non-overlapping sequences in the same analysis, which is not possible with distance-based methods, such as NJ. In addition, there is a growing concern about the validity and adequacy of both NJ and K2P for barcode analysis, especially when compared with methods like ML under the best fit evolutionary model 45 ^,^ 46 ^,^ 47. The NJ and ML trees were compared for the standard and unique alignments to evaluate consistency of the results. Confidence level was estimated for both methods as percentage recovery after 10,000 bootstraps. Putative species were inferred using the Poisson tree processes model (PTP) on the ML trees 52. These putative species are equivalent to molecular operational taxonomic units (MOTU 53) and were compared with the morphologically-identified species (OTUs).

For the BLAST approach, jMOTU 15 was used on the original, standard and unique alignments. The minimum alignment length (i.e., overlap between sequence pairs) for analysis of the original dataset was set at 100 bp. The standard alignment showed minimum overlap of ~350 bp between pairs of sequences; 400 bp was common to all sequences in the unique alignment. The BLAST filter was 85 for all analyses. The tree results, resolved MOTUs, and identified OTUs were compared for the three alignments.

**Frequency distribution of the 1,381 sequences from the original dataset by length (in base pairs).**
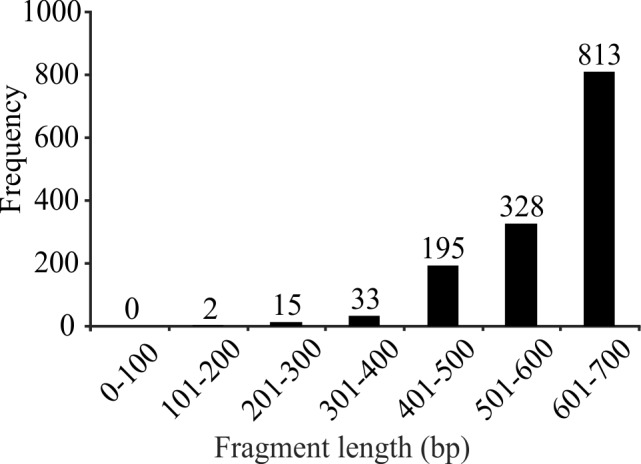
A total of 1,141 sequences (82%) fulfilled the minimum of 500 bp length definition of a gold standard barcode.

**Species-by-species analyses**

Taxa showing discrepancies between MOTUs and OTUs were selected for additional analysis, when possible based on available data, to examine possible reasons (e.g., variation among geographic areas or populations, cryptic speciation) for the observed disparities between morphological and molecular data. Analyses included parsimony haplotype networks (gene genealogies) using TCS Ver. 1.2.1 54 and calculation of *F*_ST_ distances between samples or regions using Arlequin Ver. 3.5 55.

## Results

This study reports a total of 800 new DNA barcode sequences for identified specimens of 63 species not included in previous studies. These new data were analyzed with 581 previously published sequences, yielding a total dataset of 1,381 sequences with an average length of 578.9 ± 84.3 bp (range: 105 – 658 bp); 82% of the sequences were > 500 bp (Fig. 1). The sequences originated from 195 different taxa or OTUs, including 71 genera, 37 families and 4 orders. Of the 1,381 total sequences, 1,354 belonged to the Order Calanoida (see Supplementary data S1.Alignment.fas at http://dx.doi.org/10.6084/m9.figshare.987095).

**Descriptive statistics**

The sliding window analysis of the unique alignment using the 100 bp window length showed that nucleotide diversity (π) was lower toward the 5’ end of the barcode region, but was relatively constant and higher in the half of the region toward the 3’ end (Fig. 2). For the analysis using the 10 bp window length, the results were markedly irregular, reflecting variation among different domains along the COI barcode region, with moderately conserved regions separated by highly variable ones.

**Sliding window analyses of nucleotide diversity (π) along the “unique” alignment (see Methods).**
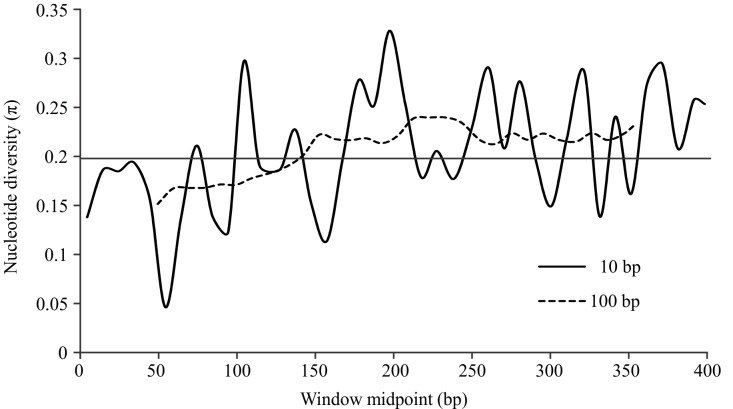
The horizontal line indicates the average π for the fragment, 0.206. Analyses with window lengths of 10 bp and 100 bp were run with a 10 bp step size; both analyses showed lower variability on the 5’ end of the amplified fragment.

Based on the unique alignment, K2P distances between species were larger than those within species (p < 0.001), but some overlap was observed and no clear barcode gap 56 was identified. Some species showed high divergences between conspecific individuals, while in other cases there were no differences between individuals of different species (see Supplementary data S2.zip at http://dx.doi.org/10.6084/m9.figshare.987095). The range of variation of distances was reduced for the standard and original alignments, but there was some overlap of within- and between-species distances, which was more pronounced when comparing higher taxonomic levels (genus and above).

Analysis of the unique alignment revealed low densities of K2P distances between individuals from 0.05 to 0.15, and very low densities between 0.08 and 0.09 (Fig. 3). Overlap of within- and between-species distances was still observed (Fig. 3). Distances within and between higher-level groups also showed overlap, although these were significantly different when analyzed using multiple U tests (p < 0.001 in all cases).

**Tree-based analysis of barcodes**

The Maximum Likelihood trees based on the unique and standard alignments showed similar results to those of the NJ trees in terms of resolution and discrimination of clades, albeit with some differences in bootstrap values (see Supplementary data S3.zip at http://dx.doi.org/10.6084/m9.figshare.987095). Overall, the ML tree showed better grouping of closely-related taxa and higher recovery of deeper nodes than the NJ analysis.

**Frequency distribution (in percentages) of Kimura-2-Parameter (K2P) distances by taxonomic level: species, genus, family, order and subclass.**
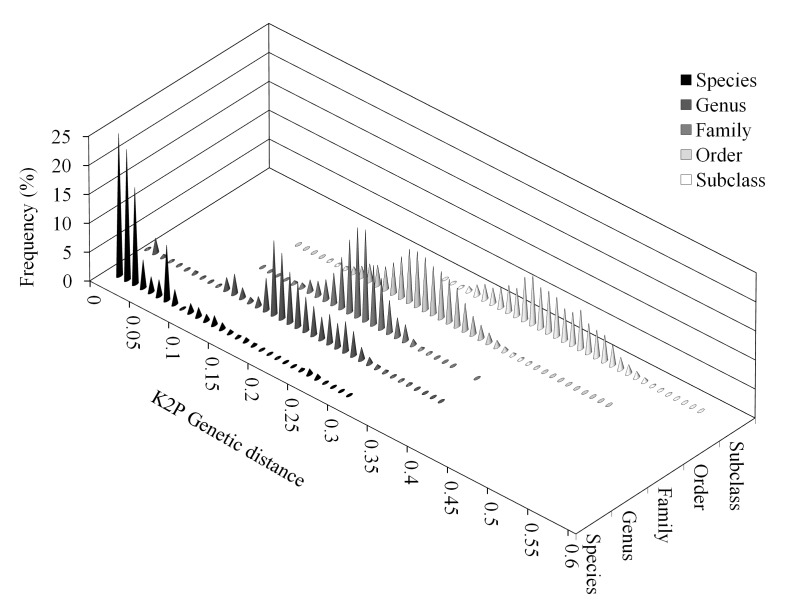
Overlap of within- and between-species distances was still observed. Distances within and between higher-level groups also showed overlap.

Automated tree-based analyses of the unique alignment resolved 227 MOTUs for the ML tree and 241 for the NJ tree. Examining the tree by eye, these MOTUs could be reduced to 65 distinct species-specific clusters, each with more than one sequence separated by short internal branches. Bootstrap recovery was > 98% (100% in most cases; see Supplementary data S3.zip). A number of taxa showed fragmentation (i.e., separation of clusters within the species grouping), indicating geographic differentiation or cryptic speciation; these clusters were identified as different putative species by the PTP analysis (Supplementary data S3.zip). In contrast, there were highly supported clades comprising sequences from different species, including species of *Calanus* (*C. helgolandicus* Claus 1863 and *C. euxinus *Huselmann 1991; *C. agulhensis* De Decker, Kaczmaruk & Marska 1991 and* C. sinicus *Brodsky 1965); *Centropages* (*C. typicus *Kröyer 1849 and *C. chierchiae *Giesbrecht 1889); *Acartia* (*A. tonsa *Dana 1849 and *A. hudsonica *Pinhey 1926); *Pleuromamma* (*P. gracilis* Claus 1863 and *P. piseki* Farran 1929); as well as a *Paracalanus* Boeck 1964 species clade.

The standard alignment identified most species with 99 - 100% bootstrap confidence; the PTP automated method identified 222 putative species from the ML tree and 277 for the NJ tree. In general, the automated tree approach failed to group conspecific individuals when the depth of sampling in that taxon was low, even though the species were grouped in a single clade with high bootstrap support in other tree-based analyses. Inclusion of additional sequences allowed better resolution of MOTUs, especially for clades showing variable or complex results (e.g., *Acartia tonsa* / *hudsonica*, *Pleuromamma gracilis* /* piseki*, and *Paracalanus* species).

**Number of MOTU inferred from the three alignments at a range of cut-offs (x-axis) expressed as percentage (relative to the mean length of each dataset) of differences between sequences.**
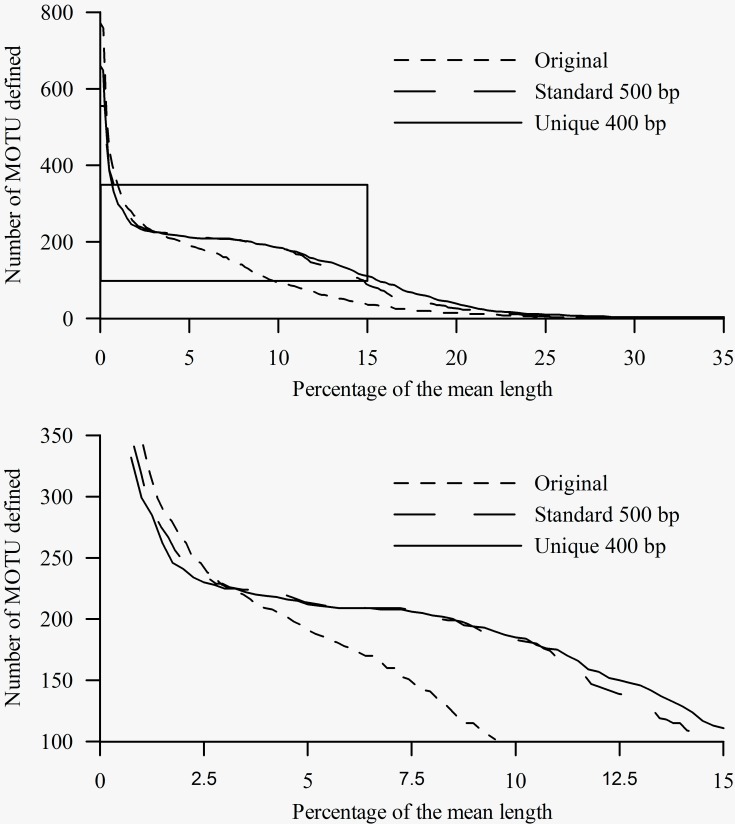
The lower panel shows in detail the box from the upper panel.

ML analysis of the original alignment yielded results that were fully comparable to those for the standard and unique alignments; PTP identified 249 putative species. Deeper nodes showed better support when additional individuals were analyzed. Even the shortest sequences (17 sequences of 105 - 288 bp; Fig. 1), were placed in the correct species clade, with no decrease in the confidence value. The automated method again yielded variable results for taxa with small numbers of individuals. In some cases, analysis of additional individuals segregated closely-related sister species into different clades, although the monophyly of the morphological species was retained in all cases. When genetic distances between conspecific individuals were moderately high (5-7 %), PTP analysis failed to identify these as a single putative species (Supplementary data S3.zip).

**BLAST analysis of barcodes**

Results of the BLAST analyses carried out in jMOTU showed more sensitivity to sequence homogeneity than did the tree-based analyses (Fig. 4). For the unique and the standard alignments, there were marked shifts resulting in the attenuation of the slope indicating a within-species MOTU threshold of 2.5 – 3 % (Fig. 4). In contrast, the original alignment did not show this shift, instead showing progressive attenuation of the slope of the curve. For the sake of argument, if we apply a 3% sequence difference threshold level for species differentiation 9, all three alignments gave similar results: 225 clusters were identified from the unique alignment, 229 from the standard, and 228 from the original alignment, although there were differences in the taxa comprising the MOTUs between the analyses. For all three alignments, the number of MOTUs detected exceeded the number of OTUs. For the unique and standard alignments, each MOTU contained only one OTU (with the same exceptions indicated for the tree analyses). However, this analysis is not equivalent to a standard BLAST analysis of a single sequence, since the constraints (minimum length, percentage, etc) are based on averages calculated for the analyzed dataset. In a standard BLAST analysis, for which these thresholds are based on the query sequence length, even the shorter sequences are properly identified.

**Best close match analysis of barcodes**

Clades or MOTUS were analyzed individually using best close match, with a primary focus on those for which there were discrepancies between MOTUs and OTUs. The results indicated that, in nearly all cases when more than one individual of the same species was included in the analyzed dataset, the closest match was the same species. Exceptions to this outcome included closely-related species pairs of *Calanus* (*C. helgolandicus* and *C. euxinus*; *C. agulhensis* and* C. sinicus*);* Centropages* (*C. typicus* and *C. chierchiae*); and *Acartia* (*A. tonsa* and *A. hudsonica*). In sum, although the MOTU/OTU concordance can be improved in comparison with automated procedures in a flexible (but subjective) fashion, total agreement between morphological and molecular species assignment methods was not possible.

**Species-by-species analyses**

Taxa showing discrepancies between MOTUs and OTUs selected for additional analysis, when possible based on available data, to examine possible reasons (e.g., variation among geographic areas or populations, cryptic speciation) for the observed disparities between morphological and molecular data, are studied in detail in Appendix 1.

## Discussion

The growing use of DNA barcodes to discriminate and identify marine animal species has included many studies on zooplankton and a number of studies of planktonic copepods (see Bucklin et al. 8 for a review). This study presents results of comparative analysis of a large dataset of 1,381 barcode sequences for 195 copepod species, including 800 new barcode sequences for 63 copepod species not included in any previous study. Evaluations include ML and NJ automated tree-based, BLAST, and “best close match” analyses of three different sequence alignments, varying the analyzed sequence domain and the numbers of individuals per species. We report here our conclusions regarding the reliability and resolution of diverse statistical approaches to species identification of planktonic marine copepods based on DNA barcodes.

The “best close match” 49 yielded the best results in terms of establishing a species threshold that avoids false positive results – even without a previously-identified and barcoded individual of the same species. Although individuals may fail to be assigned to a species, incorrect species assignments are avoided, unless the distance between two morphologically identified OTUs is zero. This analysis avoids a frequent error of NJ trees, for which individuals of different species may cluster together – albeit on long branches – with bootstrap support equal or very close to 100% when one or more of the species is missing from the analyzed barcode dataset 12 ^,^ 18.

The poor performance of the tree-based automated method compared to the others, can be attributed to the unbalanced dataset (large disparity in numbers of individuals among species) and with low coverage in many cases. This fact is known to limit the performance of PTP and other similar tree-based delimitation methods 52 . On the other hand, the failure of the PTP approach resulted in lack of power to identify the species, and not the much less desirable error of wrong species assignment.

The high levels of genetic diversity within species and the limited number of species for which DNA barcodes are available make character-based diagnosis 11 ^,^ 12 very unlikely to succeed. This approach may be appropriate for much-studied and well-defined groups of taxa, where much of variability has been characterized. Especially when a small number of nucleotide substitutions are used as taxon identifiers or as a step in a taxonomic key, accurate species identification will require complete knowledge of variability both among populations of a species and among species of the group of interest. We are still far away from this goal for marine copepods, due to their large effective population sizes (on the order of 10^8^ 57) and exceptional genetic diversity among eukaryotes 58.

Although the coincidence of these two concepts (large population sizes and exceptionally high genetic diversity) could seem counter-intuitive 59, this should be considered in light of factors inherent to the marine planktonic environment. The large distributional ranges of most species, in many cases across multiple ocean basins, might facilitate the isolation of lineages, while still allowing migration and continuous exchange of individuals across the distributional range. The short generation time for these species (usually weeks to several months, rarely multiple years) makes impossible the migration of individuals across the entire range of the species in a single or few generations. Thus, both oceanographic barriers and isolation by distance may results in population differentiation at large scales and among different ocean basins. However, if analyzed at fine scales, allele frequency differences would show continuous variation, with stronger differences at hydrographic or biogeographical barriers only 35 ^,^ 86 ^,^ 87 ^,^ 88.

One of the most powerful applications of barcoding for marine copepods is the analysis of the entire zooplankton community through high throughout DNA sequencing of environmental samples, out-performing the results obtained even by trained morphological analysts 60 . Recent technical advances allow determination of long sequences necessary for accurate identification of species in mixed assemblages. Limitations include inefficient amplification of the COI barcode region in samples containing diverse taxa resulting from variability in the amplification priming sites 61, which hinders annealing of consensus primers. However, higher affinity and amplification success rates of more conserved genes have the associated problem of under-estimating the real diversity of species in a community 62 ^,^ 63 ^,^ 64, due to low levels of sequence divergence and lack of discrimination between closely related species. The low affinity of the consensus COI barcoding primers by Folmer et al. 38 can be countered by design of suites of group-specific primers; copepod-specific primers have been designed for this purpose 20 ^,^ 65 . In the very near future, environmental barcoding approaches may employ nested sets of species- and group-specific amplification and sequencing primers and protocols to ensure reliable, accurate, cost-effective, and rapid assessments of species-level of diversity of pelagic communities, including the taxonomically complex and ecologically-important copepods.

**OTUs vs MOTUs**

Automated statistical analyses allow species identification and detection of species boundaries based on DNA barcodes 16 ^,^ 17. However, our results showed a large discrepancy between the numbers of OTUs and MOTUs (e.g., for the original alignment, 195 morphological species versus 249 / 228 putative species on the ML tree / jMOTU, respectively). Since these numbers are based on a 3% threshold for discrimination in jMOTUs, this may be due in some cases to unrecognized cryptic species. In other cases, the discrepancy may reflect strong population structuring of widely-distributed species, perhaps combined with incomplete sampling of populations across the geographic range (see discussion in Bergsten et al.^38^ ^,^ 66). Those errors could be corrected by non-automated approaches that would not be suited for larger dataset, such as the best-close match or examining the problematic clades on the tree by eye. It is not rare for marine copepods to show genetic differences over 5 % between individuals within and between populations 31 ^,^ 35. Although those cases may be easily resolved by considering the geographical reference (collection location) and/or closely-related species, detection by automated analysis is difficult without geographically and taxonomically dense and balanced sampling. In other cases, the putative species delimitation would be biased by over-sampled taxa 52. Marine planktonic copepods are excellent examples of the inherent challenges of sampling highly abundant, widely distributed populations: high spatial resolution and geographically-extensive sampling is needed for a perfect match between OTUs and MOTUs. But, despite under-sampling of intraspecific variation in the dataset analyzed here, there were no false positives (i.e., assignment of the wrong species to an individual) and the genetically closest individual to any specimen identified using a barcode almost always (with a few notable exceptions) belonged to the same species.

A criticism of metazoan barcoding is the reliance on a single gene, rather than multiple molecular markers. In fact, results obtained from additional genes do not always yield the same results, and caution is advised when using only one or few genetic markers. Additional sources of error include sample sizes, geographical coverage, and sampling bias. In sum, many problems associated with barcoding result not from the COI barcoding region, but from relying on a single molecular marker without necessary consideration for the inevitable limitations, since any gene – even very conserved ones – will have strengths and pitfalls 64. It is possible that there may be better regions for species assignment, and longer sequences do provide better accuracy and reliability 61, but our results confirm that even very short COI fragments (< 150 bp) show acceptable levels of accuracy for species identification. Further, although average COI divergence is significantly higher for deeper taxonomic levels, there is no consistent relationship between divergence and taxonomic level. COI shows marked saturation and erosion of the phylogenetic signal for deeper nodes 67.

A primary limitation of barcoding is the widespread problem of incorrect species identification in published datasets, which markedly reduces reliability and usefulness of the approach 18 ^,^ 49 ^,^ 68. This problem was detected in our dataset by comparison with data from GenBank and other public databases. In other cases, when the obvious morphological differences between the two species made misidentification unlikely, errors may result from laboratory procedures. Solutions include approaches that allow independent confirmation of identifications, e.g., inclusion of images, retention of voucher specimens for later examination, and ratings on the accuracy of taxonomists 69. Another solution is simply to continue to populate databases and increase taxon sampling densities both systematically and geographically, thus allowing recognition of errors at the time of data submission.

## Conclusions

This study presents new DNA barcode data for marine copepods (800 sequences for 63 species not previously sequenced) and reports the results of new analyses of a larger dataset (1,381 sequences for 195 copepod species). Our conclusions include recommendations to improve the accuracy and feasibility of using DNA barcodes for species identification of marine planktonic copepods, including: 1) availability of PCR and sequencing primers suited to the targeted species; 2) availability of a taxonomically-comprehensive DNA barcode database linking DNA sequences to accurately identified specimens; 3) increased density of taxon sampling; and 4) near-complete coverage for the group of interest. In particular, comprehensive databases are needed for environmental barcoding efforts (i.e., barcoding of unsorted environmental samples) that seek to characterize species-level diversity of marine zooplankton assemblages and ecosystems.

Increasingly sophisticated approaches to statistical analysis of the barcode region of the mitochondrial cytochrome c oxidase subunit I (COI) gene have resulted in new appreciation for the strengths and weaknesses of this genetic marker for species assignment of planktonic copepods. An important result is that – for all analytical approaches – accurate identification requires inclusion in the analyzed dataset of a barcode sequence for that species. The lack of a complete DNA barcode library is thus the most limiting factor for accurate and reliable discrimination and identification of species of planktonic copepods. In fact, DNA barcodes are currently available for only ~ 400 copepod species, including many parasitic and freshwater taxa. In addition, extensive coverage of species diversity is especially critical for efficient resolution on large datasets using automated methods. Fortunately, many barcoding studies have focused on ecologically important, abundant, and/or geographically widespread species and species groups, making the available DNA barcode data particularly useful. Species that are rare or geographically restricted may remain unidentifiable using barcodes for the foreseeable future.

## Figures and Tables

**TCS analysis on the Nannocalanus minor sequences.**
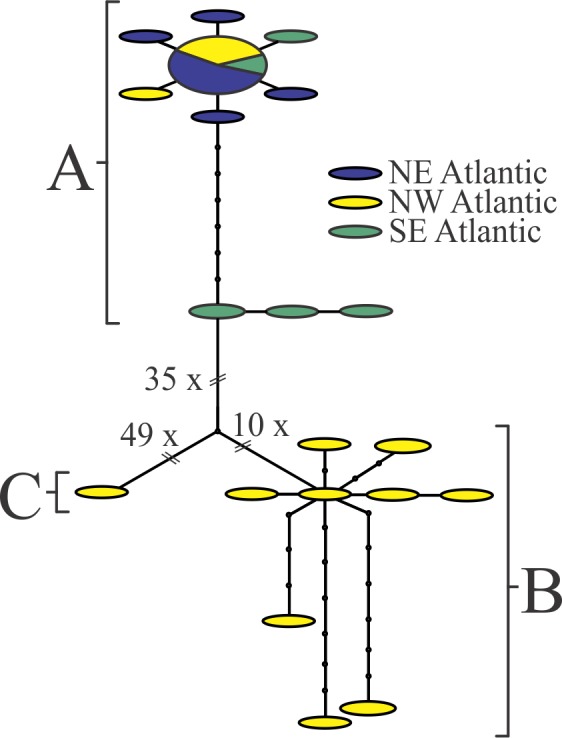
Each segment represents a mutational step, and small black circles, missing haplotypes needed to connect observed haplotypes. The size of the ovals is proportional to the number of sampled individuals with that haplotype. All sites containing ambiguous or missing data were excluded from the analysis (complete deletion; final length 485 bp). The three clusters reflect the clades identified in the tree analyses. No regional or distribution pattern could be related to these results.

**TCS analysis on the Metridia lucens sequences.**
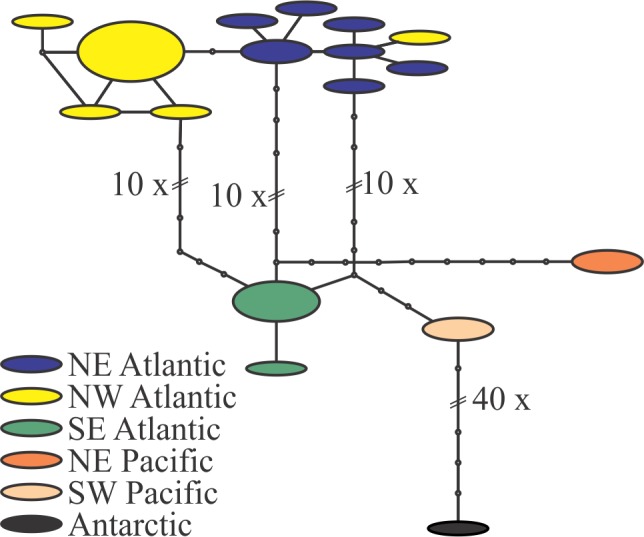
Each segment represents a mutational step, and small black circles are missing haplotypes needed to connect observed haplotypes. The size of the ovals is proportional to the number of sampled individuals with that haplotype. All sites containing ambiguous or missing data were excluded from the analysis (complete deletion; final length 502 bp). The resulting diagram reflects a strong regional pattern, with relative isolation between the different ocean basins especially in the North-South axis. The Antarctic region shows a strong isolation even from the close individuals from the Southern Hemisphere.

**TCS analysis on the Pleuromamma piseki and Pleuromamma gracilis sequences.**
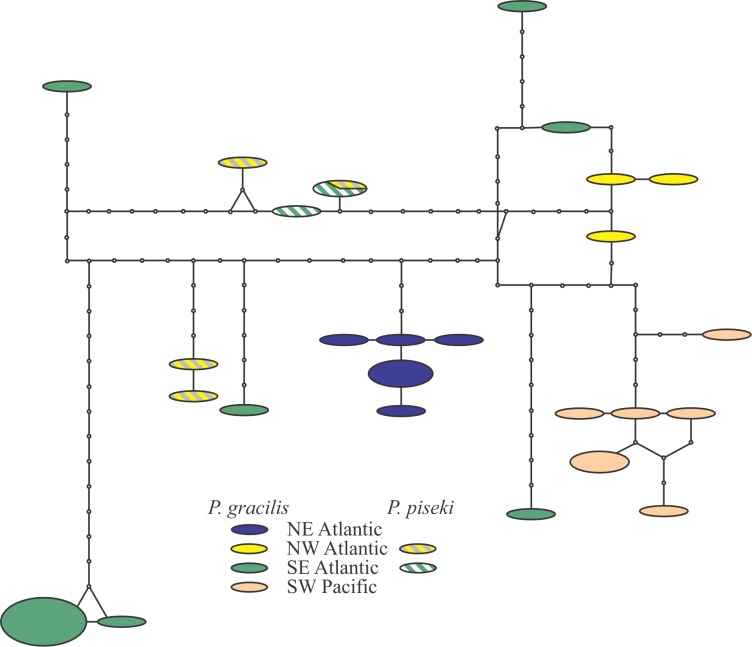
Each segment represents a mutational step, and small black circles are missing haplotypes needed to connect observed haplotypes. The size of the ovals is proportional to the number of sampled individuals with that haplotype. All sites containing ambiguous or missing data were excluded from the analysis (complete deletion; final length 502 bp). The resulting diagram reflects a strong regional pattern, with relative isolation between the different ocean basins especially in the North-South axis. The Antarctic region shows a strong isolation even from the close individuals from the Southern Hemisphere.

## Appendix 1

### Problematic taxa

Previous or ongoing publications have addressed some of the discordances between Operational Taxonomic Units (OTUs) and Molecular Operational Taxonomic Units (MOTUs) for various marine copepod taxa: *Calanus agulhensis *De Decker, Kaczmaruk & Marska 1991  / *C. sinicus* Brodsky 1965** 34 ; *Calanus helgolandicus *Claus 1863 / *C. euxinus *Huselmann 1991** 70 ^,^ 71 ^,^ 72 ; *C. lividus *Frost & Fleminger 1968  35 ; *A. tonsa* Dana 1849 /* A. hudsonica *Pinhey 1926 clades 73, Bucklin et al., unpublished; *Paracalanus* spp. 74 ^,^ 75; and *Calanoides carinatus* Krøyer 1848 (Viñas et al. unpublished).

Copepod taxa that are problematical for barcoding are most usually also problematical in classical morphological taxonomy (e.g., *Acartia,* *Paracalanus*, *Nannocalanu*s). These groups show combined genetic, ecological, biogeographical and morphological complexities that must be resolved to define and delimit species boundaries, improve our understanding of marine zooplankton community ecology, and examine species-level responses to environmental change 13 . Several examples of such taxa are described here:

*Nannocalanus minor* Claus 1863

*Nannocalanus minor* barcodes form a cluster comprising three clades in both the tree-based (Supplementary data S3.zip) and TCS analysis (Fig. 1 in Appendix). The sequence variation did not show any geographical pattern; all three clades contained sequences from the NW Atlantic and in some cases were found in the same sample. Cluster A included a number of individuals with identical sequences collected from the NE and SE Atlantic; within each clade, individuals differed by < 1.5% on average; between clades, differences ranged from 9.2 - 14.3% (Table 1). In addition, Φ_ST_ (*F*_ST_ distance under the K2P model) = 0.95 between clades A and B (P value < 0.0001 after 10,000 permutations); Φ_ST_ distances were not calculated for Clade C, which included a single sequence. Morphological analyses of two females from Clade A and B, respectively indicate different numbers of teeth on the inner edge of the basipodite 1 of P5: the Clade A individual showed 11 denticles, compared to 16 for Clade B. Individual variability is known to be high for this character, and either of the two described *Nannocalanus* species may exhibit 16 teeth. However, 11 teeth on the P5 B1 is outside the known range for this character for *N. minor* *sensu stricto* 3 ^,^ 76, suggesting potential reproductive isolation among the three clades, which may be sympatric, cryptic species 77.

*Mesocalanus tenuicornis* Dana 1849

Sequences for *Mesocalanus tenuicornis* clustered with low bootstrap support (Supplementary data S3.zip) and showed two clades, the SW Pacific and the NE Atlantic. Two other divergent sequences from SW Pacific and SE Atlantic were consistent with the jMOTU results that resolved four independent MOTUs. The divergence between these four potential clades comprised of two groups and two individuals ranged from 11.6 - 18.8% (average = 15.6 ± 3.2% S.D.), with individuals collected from the same location (e.g., AF332788 and AF462316) showing 16.7% COI divergence. Compared to another COI sequence for this species from GenBank (AB379998), genetic distances obtained here were in the same range (15.8 - 18.9%). Although some differences may result from geographic differentiation of regional populations, the presence of very divergent clades indicates the possible presence of cryptic species within this taxon.

*Euterpina acutifrons *Dana 1848

Tree-based and BLAST analysis of the harpacticoid copepod *Euterpina acutifrons* yielded two clusters corresponding to the NE (Bay of Biscay) and NW (Mid-Atlantic Bight) Atlantic. Within clades, differences ranged from 0.0 - 0.5%, while between-clade differences averaged 12% (range 11.8 – 12.6%; Supplementary data S2.zip and S3.zip). Although planktonic, *E. acutifrons* is rarely found in the open ocean 3 and naupliar stages are linked to the hyperbenthic environment 78, suggesting limited dispersal across and among ocean basins. Other harpacticoid copepods showed similarly large genetic divergences among geographic populations, including a species found in tidal pools and benthic environments 36, although open-ocean harpacticoid species showed lower divergences among ocean basin than in our study 31. These considerations, together with our small sample size, prevent conclusions of whether the observed differences reflect strong divergences among regional populations of a single species or the presence of a cryptic species complex within *E. acutifrons*.

*Centropages typicus* Kröyer 1849 and *Centropages chierchiae* Giesbrecht 1889

These two species co-occur throughout most of their distributions in the Atlantic Ocean 3. Subtle morphological differences discriminate the species 79  and many individuals exhibit ‘intermediate forms’ (L.B.B. unpubl. data.; Nieves Rodriguez Garcia, Oviedo, pers. comm.). Morphological variation has been widely described for C. typicus; comparative analyses showed no genetic basis for the morphological variation 32. In our study, genetic distances between individuals of both species from both NW and NE Atlantic (Bay of Biscay) were lower than 4%, and very similar within- and between-species (Table 2). In addition, an ‘intermediate-form’ individual showed the same range of variation as both species. In light of both morphological and molecular data, there is significant doubt about the validity of the distinction of *C. chierchiae* Giesbrecht, 1889 from C. typicus Kröyer, 1849.

*Calocalanus contractus *Farran 1926

Two very similar sequences (differing by < 1%) were found for *Calocalanus contractus*; a third sequence differed by 9% from these two; the corresponding amino acid sequence was identical for these three individuals. These three sequences differed from other *Calocalanus* species by 17.4% to 18.4%. The 9% difference is just slightly smaller to that between sister species within this genus 74, but some other copepod species show intra-species distances in this range 35. A detailed study combining morphology, taxonomy and barcoding is needed for the ~50 species described species 3, including many poorly-described species with small size and complex ornamentation.

*Acartia tsuensis* Ito 1956 and *A. tonsa *Dana 1849 /* A. hudsonica *Pinhey 1926

Three differentiated clades were detected within *Acartia tsuensis*. One was collected from Momoshima Island, Japan, while the other two were collected from fish ponds in the Philippines. Genetic distances within the clades were below 2%, while distances between the clades were equal or larger than 19% (average = 19.6%, SD = 0.4%), probably reflecting genetic isolation between the clades and indicating cryptic speciation.

For *A. tonsa* and* A. hudsonica*, individuals of both species were mixed within each clade; individuals from the NE Pacific samples (identified as two *A. tonsa* and one *A. hudsonica* individuals) clustered together. Cryptic speciation among geographically-close or sympatric populations has been described for other species of *Acartia*, likely driven by limited dispersal among populations and the effects of mesoscale and local environmental conditions. Alternatively, complex phylogeographic patterns might reflect high levels of genetic differentiation among conspecific populations 33 ^,^ 37 ^,^ 80 ^,^ 81.

*Metridia lucens * Boeck 1864

*Metridia lucens* is a cosmopolitan species that showed significant genetic differentiation among the six geographic regions analyzed: NW Atlantic (19 individuals), NE Atlantic (10), SW Pacific (4), NE Pacific (5) and Antarctic (1). No haplotypes were shared between ocean basins (Fig. 2 in Appendix), and genetic distances between regions were on average ten-times that obtained within regions (Table 3; the Antarctic individual was excluded from analysis). Φ_ST_ distances (under K2P model) indicated high and significant isolation between NW and NE Atlantic regions (Table 4), although this may result partly from small sample sizes bias (Fig. 2 in Appendix). It is possible that *Metridia lucens*, which exhibits strong diel vertical migration, may experience lower exchange among populations and show strong regional-scale genetic structure. The Antarctic sample showed a highly divergent sequence, ~8% different from other* M. lucens* individuals, which is similar to results obtained with larger sample sizes by Stupnikova et al. 82. As comparison, genetic distances between *M. lucens* and the closely related *M. pacifica* Brodsky 1950 ranged from 12.6% - 14.4%. Consequently, genetic variation among Antarctic *M. lucens* samples may reflect either recent speciation or strong regional isolation.

*Pleuromamma gracilis* Claus 1863 and *P. piseki* Farran 1929

The clade containing *Pleuromamma gracilis* and* P. piseki* was recovered with 100% bootstrap support, but the species formed a number of clusters and MOTUs. A detailed analysis of these two taxa showed a complex pattern, with strong regional-scale genetic structuring of *P. gracilis*, but no clear isolation from *P. piseki* (Fig. 3 in Appendix; Table 5). *Pleuromamma gracilis* from the SE Atlantic showed a number of highly divergent haplotypes, but still no amino acid substitutions (Fig. 3 in Appendix). Regional-scale genetic structure and the presence of highly-divergent rare haplotypes may be related to the large effective population size of cosmopolitan marine copepods 35 ^,^ 83. In contrast, *P. piseki* did not show strong spatial structure and lacked genetic divergence from* P. gracilis* (Table 5). Similar results have been recently 84 reported for the same species complex for other mitochondrial markers .Clearly, integrated molecular and morphological analysis of both species throughout their distributional ranges is needed to clarify both the status of the species and their population structure.

*Clausocalanus* Giesbrecht 1888 species

All 13 described species of *Clausocalanus* ^84^ 85 are represented in the database, including multiple individuals from different ocean basins. All species clustered together in all analyses (Supplementary file S3.zip). Interestingly, two individuals collected from Sagami Bay (Japan) and identified as *C. arcuicornis* Dana 1849 (by L.B.B.) showed morphological oddities and were discriminated as a distinct clade with 12% or higher divergence from any of *C. arcuicornis* (or any other *Clausocalanus*) individuals. Since no intact individuals remained from the sample, a detailed morphological analysis cannot be done and we can only speculate that these individuals may represent either of two *Clausocalanus* that are* incertae sedis* (*C. latipes* T. Scott 1894 and *C. dubius *Brodsky 1950), or very divergent individuals of *C. arcuicornis*.

### Figures and Tables

**Table 1. Average, standard deviation, maximum and minimum base differences per site (p-distance) within and between sequences of three clades of Nannocalanus minor.**

	Within A	Within B	A *vs* B	B* vs* C	A* vs* C
Average	0.0044	0.0138	0.1170	0.1273	0.1403
St. Dev.	0.0060	0.0078	0.0074	0.0035	0.0016
Max.	0.0178	0.0258	0.1282	0.1322	0.1433
Min.	0.0000	0.0000	0.0928	0.1216	0.1367

**Table 2. Average, standard deviation, maximum and minimum base differences per site (p-distance) within and between Centropages chierchiae and C. typicus, and between these species and the intermediate forma.**

	*C. chierchiae *	* C. typicus*	ch. *vs* ty.	int. *vs* pure
Average	0.0127	0.0216	0.0160	0.0252
St. Dev.	0.0036	0.0123	0.0103	0.0067
Max.	0.0156	0.0362	0.0317	0.0362
Min.	0.0087	0.0035	0.0035	0.0173

**Table 3. Average, standard deviation, maximum and minimum base differences per site (p-distance) between the Metridia lucens individuals within the locations, between all locations excluding the Antarctic, and those compared to the Antarctic individual.**

	NE Atlantic	NW Atlantic	SE Atlantic	SW Pacific	NE Pacific	Between	*Vs* Antarctic
Average	0.0024	0.0016	0.0003	0.0000	0.0000	0.0236	0.0816
St. Dev.	0.0014	0.0028	0.0006	0.0000	0.0000	0.0132	0.0063
Max.	0.0047	0.0185	0.0016	0.0000	0.0000	0.0454	0.0965
Min.	0.0000	0.0000	0.0000	0.0000	0.0000	0.0016	0.0717

**Table 4. ΦST distances (K2P model, lower diagonal, and p-value (upper diagonal) between locations. Significant results after Bonferroni correction are indicated in bold.**

	NE Atlantic	NW Atlantic	SE Atlantic	SW Pacific	NE Pacific
NE Atlantic		<0.0001	<0.0001	0.0006	0.0002
NW Atlantic	**0.763**		<0.0001	<0.0001	<0.0001
SE Atlantic	**0.978**	**0.969**		0.001	0.0002
SW Pacific	**0.980**	**0.968**	**0.868**		0.008
NE Pacific	**0.986**	**0.977**	**0.983**	1.000	

**Table 5. Average, standard deviation, maximum and minimum base differences per site (p-distance) within and between the P. piseki and P. gracilis individuals, and within P. gracilis locations with N > 10 individuals.**

	*P. piseki*	*P. gracilis*	Between	NE Atlantic	SE Atlantic	SW Pacific
Average	0.0321	0.0611	0.0662	0.0030	0.0401	0.0088
St. Dev.	0.0249	0.0359	0.0177	0.0019	0.0452	0.0059
Max.	0.0601	0.1030	0.0934	0.0064	0.1030	0.0218
Min.	0.0000	0.0000	0.0277	0.0000	0.0000	0.0016
